# TAVR for Pure Aortic Regurgitation: A Patient-Specific Simulation-Based Feasibility Study

**DOI:** 10.1016/j.jacasi.2025.12.012

**Published:** 2026-03-03

**Authors:** Yu Mao, Mengen Zhai, Ping Jin, Yanyan Ma, Haotian Gao, Chunli Tao, Yingying Gou, Yang Liu, Jian Yang

**Affiliations:** a Institute of Science and Technology for Brain-Inspired Intelligence, Fudan University, Shanghai, China; b Department of Cardiovascular Surgery, Xijing Hospital, Xi’an, Shaanxi, China; c Chengdu Silara Medtech Inc, Chengdu, Sichuan, China; d Department of Clinical Research, Xi’an Make Medical Technology Co, Ltd, Xi’an, Shaanxi, China

**Keywords:** 3D printing, aortic regurgitation, computational fluid dynamics, Silara system, transcatheter aortic valve replacement

Aortic regurgitation (AR) affects up to 10% of older adults.^1^ Surgical aortic valve replacement (SAVR) is standard for severe AR, but many patients are ineligible because of frailty, impaired left ventricular (LV) function, or major comorbidities.^2^ Although transcatheter aortic valve replacement (TAVR) is well established for aortic stenosis,^3^ its use in pure AR is challenging. Large, elliptical annuli or absent leaflet calcification, and dilated aortic roots compromise transcatheter heart-valve anchoring.^4^ Patient-specific 3-dimensional (3D) printing^5^ and computational fluid-dynamics (CFD) simulation^6^ have emerged as valuable planning tools for complex anatomies. Building on these technologies, we evaluated the novel, fully retrievable Silara TAVR system in high-risk patients with pure AR.

This prospective single-center study (NCT02917980) was performed at the Department of Cardiovascular Surgery, Xijing Hospital, between December 2022 and August 2023. Six consecutive patients with symptomatic severe pure AR were enrolled. All were considered inoperable or at high surgical risk by a multidisciplinary heart team. The cardiac team decision was based on a comprehensive assessment integrating factors not fully captured by the Society of Thoracic Surgeons score, including severe frailty, major noncardiac comorbidities, and complex aortic root anatomy typical of pure AR that significantly increased the technical risk of surgical aortic valve replacement. All patients met the key inclusion criteria (age ≥60 years, NYHA functional class ≥II or higher, life expectancy >1 year). Exclusion criteria included extreme annular dimensions, recent acute cardiovascular events, or severe LV dysfunction. The study followed the Declaration of Helsinki and was approved by the Xijing Hospital Ethics Committee (KY-20192138-C-1), and all participants provided written informed consent.

The TAVR system uses a reaction-fixed design featuring dual annular fixation zones, providing secure anchoring without requiring rapid ventricular pacing (Figure 1A). Valve deployment is achieved by injecting radiopaque exchange solution through a central positioning catheter, which expands the anchoring structures to engage the native annulus. Once proper placement is confirmed, the expansion medium is replaced with the polymer, which solidifies to provide long-term radial support. The delivery system accommodates complete retrieval and repositioning before final deployment.

**Figure 1:**
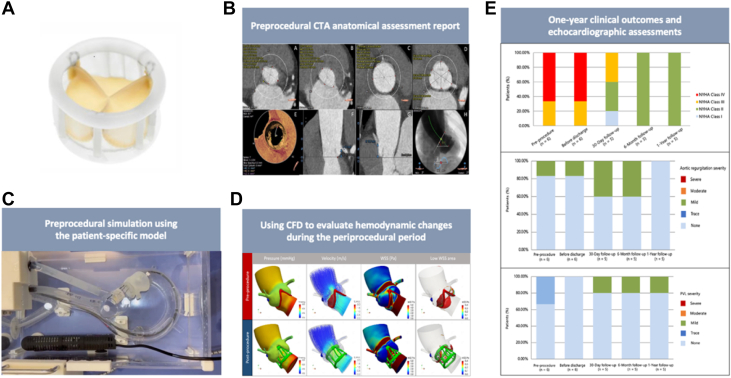
The TAVR System for Pure AR (B) Preprocedural imaging, combined with (C) 3-dimensional (3D) printing and (D) computational fluid dynamics (CFD)-based personalized simulation for comprehensive assessment, enables transfemoral transcatheter aortic valve replacement (TAVR) using (A) the system. (E) 1-year follow-up indexes confirm the safety and efficacy in patients with pure aortic regurgitation. CTA = computed tomography angiography.

All patients underwent preprocedural contrast-enhanced computed tomography angiography and transthoracic echocardiography (TTE) (Figure 1B). Patient-specific aortic root models were created using a Stratasys PolyJet 850 printer with tissue-mimicking materials (Figure 1C). CFD analysis (Figure 1D) guided optimal valve sizing and implantation depth. TAVR was performed under general anesthesia with initial device placement in the LV, followed by precise annular positioning using 3 tensioning sutures. Clinical outcomes were adjudicated according to Valve Academic Research Consortium-3 definitions^7^ at baseline, discharge, 30 days, 6 months, and 1 year. Statistical analyses employed SPSS 26.0. Paired Student’s *t*-tests were used to compare baseline and follow-up measurements; *P* < 0.05 was considered significant.

All patients were women (mean age 69.5 ± 2.2 years, mean Society of Thoracic Surgeons score 3.1% ± 1.8%). All cases had severe AR, with mean LV ejection fraction 54.8% ± 5.0%, and mean annulus diameter 23.7 ± 1.2 mm (only patient #2 had mild calcification). Preprocedural 3D printing and CFD simulations predicted valves sizes (3 patients for 25 mm, 2 patients for 27 mm, 1 patient for 29 mm) and implantation depth (9.5 ± 2.7 mm vs 9.2 ± 2.9 mm) that closely matched intraprocedural findings.

Device success was 100% by Valve Academic Research Consortium-3 criteria. Mean procedural time was 98.3 ± 22.5 minutes; mean contrast volume was 60.0 ± 6.3 mL. One patient (#4) required valve retrieval and redeployment because of initial malposition, no sequelae. Immediate postprocedure transesophageal echocardiography showed a mean transvalvular gradient of 5.7 ± 1.0 mm Hg and mean annular area of 2.0 ± 0.3 cm^2^; 4 of 6 patients (66.7%) had no or only trace paravalvular leak (PVL).

Predischarge, no patient experienced in-hospital mortality or major adverse cardiovascular and cerebrovascular events, and TTE demonstrated significant reverse LV remodeling in all patients, as evidenced by reductions in LV end-systolic volume (34.6 ± 6.7 mL vs 42.2 ± 13.6 mL; *P* < 0.001) and LV end-diastolic volume (77.0 ± 6.1 mL vs 93.7 ± 23.0 mL; *P* < 0.001).

The median follow-up of 6 patients was 12 months, with 5 patients completing at least full 1-year follow-up (354-390 days). There were no deaths, strokes, myocardial infarctions, major bleeding events, or new permanent pacemaker implantations. At 30 days, all 5 follow-up patients had symptomatic improvement, 3 (60.0%) achieving NYHA functional class I/II. At 6 months and 1 year, all patients remained in NYHA functional class II, with sustained improvements in exercise tolerance and dyspnea (Figure 1E). Follow-up TTE showed preserved valve function. At 30 days and 6 months, 3 (60%) of 5 follow-up patients had no residual AR/PVL, and 2 (40%) had mild regurgitation. At 1 year, only 1 patient (20%) had mild PVL, and no AR recurrence was noted. No significant changes were observed in transvalvular gradient, annular area, or valve depth between discharge and 1 year. Improvements in LV function persisted: LV ejection fraction: 60.4% ± 4.0% vs 54.8% ± 5.0% pre-TAVR, LV end-systolic volume: 28.4 ± 4.7 mL vs 42.2 ± 13.6 mL, and LV end-diastolic volume: 71.2 ± 8.4 mL vs 93.7 ± 23.0 mL (*P <* 0.001).

This first-in-human study shows the TAVR system safely and effectively treats pure AR in high-risk patients. Personalized 3D printing and CFD modeling were instrumental in precise valve sizing and positioning. The value lies not in tool novelty, but in a device-specific simulation protocol that optimized implantation parameters for safe use. Notably, paravalvular leak was low likely because of the device’s unique polymer-based solidification. Unlike traditional skirt-based designs, this technology adapts to native annular anatomy, potentially offering an advantage in pure AR. Limitations include small sample size, single-center design, and no simulation-free control arm; larger multicenter trials with longer follow-up are needed to validate findings. We have standardized protocols for efficiency. Future work should focus on semiautomated pipelines to boost clinical adoption of this tailored planning, especially for complex cases or novel devices.

TAVR with the system is feasible, safe, and promising for high-risk patients with pure aortic regurgitation. Incorporating patient-specific 3D printing and CFD simulation into preprocedural planning may further improve procedural safety and long-term outcomes.

### Availability of data and material

The original contributions presented in the study are included in the paper/supplemental material. Further inquiries can be directed to the corresponding author.

## Funding Support and Author Disclosures

This work was supported by the Development and Transformation of New Technology and Construction of Precision Diagnosis and Treatment System for Transcatheter Interventional Diagnosis and Treatment of Structural Heart Diseases (2022YFC2503400); National Natural Science Foundation (82370375); Research on Key Techniques of Minimally Invasive Treatment for Valvular Heart Diseases (2023-YBSF-105); Xijing Hospital Booster Foundation (XJZT24LY42); and Safety and Efficacy of 3D Printing in Transcatheter Aortic Valve Replacement: A National Multicenter, Prospective Study Program (XJZT24LY42). The authors have reported that they have no relationships relevant to the contents of this paper to disclose.

## References

[1] Elder D.H., Wei L., Szwejkowski B.R. (2011). The impact of renin-angiotensin-aldosterone system blockade on heart failure outcomes and mortality in patients identified to have aortic regurgitation. J Am Coll Cardiol.

[2] Iung B., Baron G., Butchart E.G. (2003). A prospective survey of patients with valvular heart disease in Europe:The Euro Heart Survey on Valvular Heart Disease. Eur Heart J.

[3] Forrest J.K., Ramlawi B., Deeb G.M. (2021). Transcatheter aortic valve replacement in low-risk patients with bicuspid aortic valve stenosis. JAMA Cardiol.

[4] Sawaya F.J., Deutsch M.A., Seiffert M. (2017). Safety and efficacy of transcatheter aortic valve replacement in the treatment of pure aortic regurgitation in native valves and failing surgical bioprostheses: results from an international registry study. JACC Cardiovasc Interv.

[5] Wang D.D., Qian Z., Vukicevic M. (2021). 3D printing, computational modeling, and artificial intelligence for structural heart disease. JACC Cardiovasc Imaging.

[6] Dowling C., Bavo A.M., Faquir N.E. (2019). Patient-specific computer simulation of transcatheter aortic valve replacement in bicuspid aortic valve morphology. Circ Cardiovasc Imaging.

[7] Généreux P., Piazza N., Alu M.C. (2021). Valve Academic Research Consortium 3:updated endpoint definitions for aortic valve clinical research. Eur Heart J.

